# 3-[5-(4-Fluoro­phen­yl)-1,3,4-thia­diazol-2-yl]-2-(4-methoxy­phen­yl)-1,3-thia­zolidin-4-one

**DOI:** 10.1107/S1600536808019089

**Published:** 2008-06-28

**Authors:** Li-He Yin, Rong Wan, Feng Han, Bin Wang, Jin-Tang Wang

**Affiliations:** a Department of Applied Chemistry, College of Science, Nanjing University of Technology, No. 5 Xinmofan Road, Nanjing, Nanjing 210009, People’s Republic of China

## Abstract

The title compound, C_18_H_14_FN_3_O_2_S_2_, was synthesized by the reaction of 5-(4-fluoro­phen­yl)-*N*-(4-methoxy­benzyl­idene)-1,3,4-thia­diazol-2-amine and mercaptoacetic acid. The thia­zolidinone ring adopts a twist conformation. The 4-methoxy­phenyl ring is almost perpendicular to the thia­diazole ring, making a dihedral angle of 88.4 (3)°. The 4-fluoro­phenyl ring is nearly coplanar with the thia­diazole ring, the dihedral angle being 6.8 (3)°. The crystal structure involves C—H⋯N, C—H⋯O and C—H⋯S hydrogen bonds.

## Related literature

For related literature, see: Arun *et al.* (1999 ▶); Chen *et al.* (2000 ▶); Kidwai *et al.* (2000 ▶); Vicentini *et al.* (1998 ▶); Wasfy *et al.* (1996 ▶); Allen *et al.* (1987 ▶).
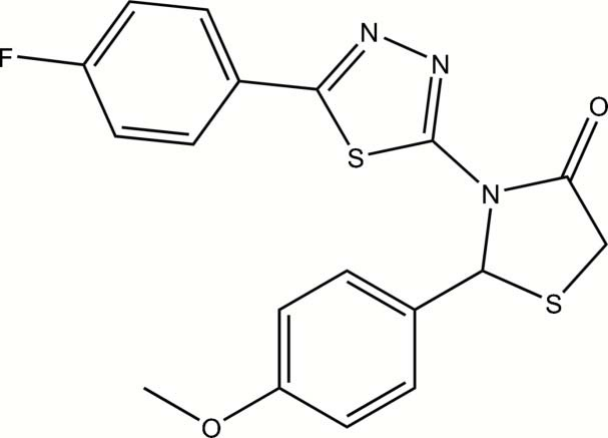


## Experimental

### 

#### Crystal data

C_18_H_14_FN_3_O_2_S_2_
*M* *_r_* = 387.44
Triclinic, 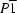

*a* = 6.4550 (13) Å
*b* = 8.9200 (18) Å
*c* = 16.483 (3) Å
α = 75.78 (3)°
β = 82.44 (3)°
γ = 71.11 (3)°
*V* = 869.0 (3) Å^3^
*Z* = 2
Mo *K*α radiation
μ = 0.34 mm^−1^
*T* = 298 (2) K
0.10 × 0.05 × 0.05 mm

#### Data collection

Enraf–Nonius CAD-4 diffractometer
Absorption correction: ψ scan (North *et al.*, 1968 ▶) *T*_min_ = 0.967, *T*_max_ = 0.984
3421 measured reflections
3120 independent reflections
2054 reflections with *I* > 2σ(*I*)
*R*_int_ = 0.023
3 standard reflections every 200 reflections intensity decay: none

#### Refinement

*R*[*F* ^2^ > 2σ(*F* ^2^)] = 0.070
*wR*(*F* ^2^) = 0.186
*S* = 1.00
3120 reflections
229 parameters
48 restraints
H-atom parameters constrained
Δρ_max_ = 0.41 e Å^−3^
Δρ_min_ = −0.52 e Å^−3^

### 

Data collection: *CAD-4 Software* (Enraf–Nonius, 1989 ▶); cell refinement: *CAD-4 Software*; data reduction: *XCAD4* (Harms & Wocadlo, 1995 ▶); program(s) used to solve structure: *SHELXS97* (Sheldrick, 2008 ▶); program(s) used to refine structure: *SHELXL97* (Sheldrick, 2008 ▶); molecular graphics: *SHELXTL* (Sheldrick, 2008 ▶); software used to prepare material for publication: *SHELXTL*.

## Supplementary Material

Crystal structure: contains datablocks global, I. DOI: 10.1107/S1600536808019089/at2579sup1.cif

Structure factors: contains datablocks I. DOI: 10.1107/S1600536808019089/at2579Isup2.hkl

Additional supplementary materials:  crystallographic information; 3D view; checkCIF report

## Figures and Tables

**Table 1:** Hydrogen-bond geometry (Å, °)

*D*—H⋯*A*	*D*—H	H⋯*A*	*D*⋯*A*	*D*—H⋯*A*
C6—H6*A*⋯N1	0.93	2.60	2.917 (9)	101
C8—H8*A*⋯O2^i^	0.98	2.52	3.233 (7)	129
C14—H14*A*⋯S2	0.93	2.74	3.146 (6)	107
C18—H18*A*⋯N3	0.93	2.57	2.881 (7)	100

Symmetry code: (i) 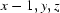.

**Fig. 1.**
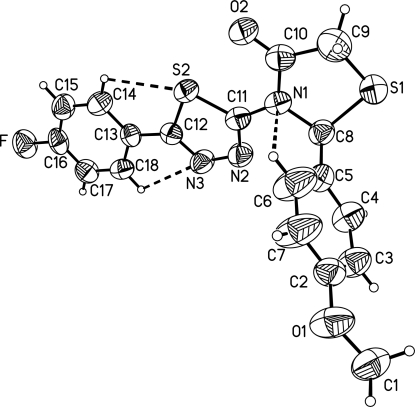
A view of the molecular structure of (I). Displacement ellipsoids are drawn at the 50% probability level. Dashed lines indicate intramolecular C—H···S and C—H···N hydrogen bonding interactions.

**Fig. 2.**
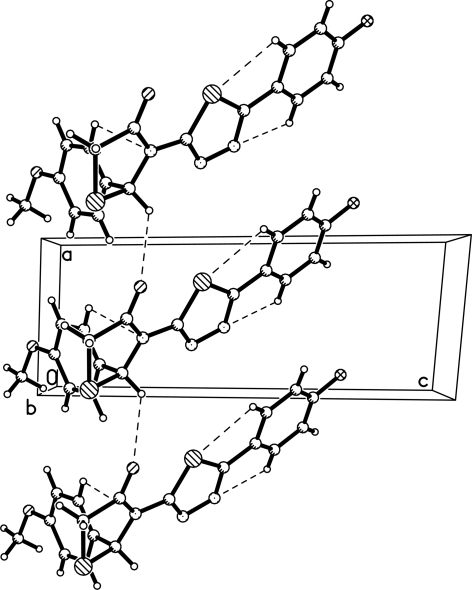
A packing diagram for (I). Dashed lines indicate intramolecular C—H···S and C—H···N, and intermolecular C—H···O hydrogen bonding interactionss.

## supplementary crystallographic information

### Comment

1,3,4-Thiadiazole derivatives containing the thiazolidinone unit are of great interest because of their chemical and pharmaceutical properties. Some derivatives have fungicidal activities and exhibit certain herbicidal activities (Chen *et al.*, 2000; Kidwai *et al.*, 2000; Vicentini *et al.*, 1998). Some show insecticidal activities (Arun *et al.*, 1999; Wasfy *et al.*, 1996).

We report here the crystal structure of the titled compound, (I). The molecular strucutre of (I) is shown in Fig.1. In this structure, the thiazolidinone adopts a twist conformation, the dihedral angle between the C9/S1/C10 and C9/N1/C10 is 15.5 (7)°. The thiadiazole ring is an aromatic heterocyclic ring, all atoms are in the same plane. The angle between the thiadiazole ring and the 4-fluorophenyl ring is 6.8 (3)°. The 4-methoxyphenyl ring is nearly perpendicular to the thiadiazole ring, with the dihedral angle being 88.4 (3)°. There are intramolecular C—H···S and C—H···N hydrogen bonding interactions in the molecule structure. In the crystal structure, intermolecular C—H···O hydrogen bonding interactionss link the molecules (Table 1 and Fig. 2).

### Experimental

5-(4-Fluorophenyl)-*N*-(4-methoxybenzylidene)-1,3,4-thiadiazol -2-amine(5 mmol) and mercapto-acetic acid (5 mmol) were added in toluene (50 ml). The water was removed by distillation for 5 h. The reaction mixture was left to cool to room temperature, filtered, and the filter cake was crystallized from acetone to give pure compound (I) [m.p. 341–345 K]). Crystals of (I) suitable for X-ray diffraction were obtained by slow evaporation of an acetone solution.

### Refinement

All H atoms were positioned geometrically, with C—H = 0.98, 0.97, 0.96 and 0.93 Å for methine, methylene, methyl and aromatic H atoms, respectively, and constrained to ride on their parent atoms, with *U*_iso_(H) = *xU*_eq_(C), where *x *= 1.5 for methyl H atoms and *x *= 1.2 for all other H atoms.

### Crystal data

**Table:**

C_18_H_14_FN_3_O_2_S_2_	*Z* = 2
*M**_r_* = 387.44	*F*_000_ = 400
Triclinic, *P*1	*D*_x_ = 1.481 Mg m^−^^3^
Hall symbol: -P 1	Melting point = 341–345 K
*a* = 6.4550 (13) Å	Mo *K*α radiation λ = 0.71073 Å
*b* = 8.9200 (18) Å	Cell parameters from 25 reflections
*c* = 16.483 (3) Å	θ = 9–12º
α = 75.78 (3)º	µ = 0.34 mm^−^^1^
β = 82.44 (3)º	*T* = 298 (2) K
γ = 71.11 (3)º	Block, colourless
*V* = 869.0 (3) Å^3^	0.10 × 0.05 × 0.05 mm

### Data collection

**Table:**

Enraf–Nonius CAD-4 diffractometer	*R*_int_ = 0.023
Radiation source: fine-focus sealed tube	θ_max_ = 25.2º
Monochromator: graphite	θ_min_ = 1.3º
*T* = 298(2) K	*h* = −7→7
ω/2θ scans	*k* = −10→10
Absorption correction: ψ scan(North *et al.*, 1968)	*l* = 0→19
*T*_min_ = 0.967, *T*_max_ = 0.984	3 standard reflections
3421 measured reflections	every 200 reflections
3120 independent reflections	intensity decay: none
2054 reflections with *I* > 2σ(*I*)	

### Refinement

**Table:**

Refinement on *F*^2^	Secondary atom site location: difference Fourier map
Least-squares matrix: full	Hydrogen site location: inferred from neighbouring sites
*R*[*F*^2^ > 2σ(*F*^2^)] = 0.070	H-atom parameters constrained
*wR*(*F*^2^) = 0.186	*w* = 1/[σ^2^(*F*_o_^2^) + (0.05*P*)^2^ + 2.5*P*] where *P* = (*F*_o_^2^ + 2*F*_c_^2^)/3
*S* = 1.00	(Δ/σ)_max_ < 0.001
3120 reflections	Δρ_max_ = 0.41 e Å^−^^3^
229 parameters	Δρ_min_ = −0.52 e Å^−^^3^
48 restraints	Extinction correction: none
Primary atom site location: structure-invariant direct methods	

### Special details

**Table:**

Geometry. All e.s.d.'s (except the e.s.d. in the dihedral angle between two l.s. planes) are estimated using the full covariance matrix. The cell e.s.d.'s are taken into account individually in the estimation of e.s.d.'s in distances, angles and torsion angles; correlations between e.s.d.'s in cell parameters are only used when they are defined by crystal symmetry. An approximate (isotropic) treatment of cell e.s.d.'s is used for estimating e.s.d.'s involving l.s. planes.
Refinement. Refinement of *F*^2^ against ALL reflections. The weighted *R*-factor *wR* and goodness of fit *S* are based on *F*^2^, conventional *R*-factors *R* are based on *F*, with *F* set to zero for negative *F*^2^. The threshold expression of *F*^2^ > σ(*F*^2^) is used only for calculating *R*-factors(gt) *etc*. and is not relevant to the choice of reflections for refinement. *R*-factors based on *F*^2^ are statistically about twice as large as those based on *F*, and *R*- factors based on ALL data will be even larger.

### Fractional atomic coordinates and isotropic or equivalent isotropic displacement parameters (Å^2^)

**Table:**

	*x*	*y*	*z*	*U*_iso_*/*U*_eq_	
S1	0.1232 (3)	1.32787 (19)	0.18123 (10)	0.0698 (5)	
S2	0.7085 (2)	0.94841 (16)	0.40097 (8)	0.0525 (4)	
F	1.1681 (6)	0.3585 (4)	0.7202 (2)	0.0868 (11)	
O1	0.3391 (7)	0.6926 (6)	0.0237 (3)	0.0873 (14)	
O2	0.6785 (6)	1.2181 (5)	0.2766 (2)	0.0669 (11)	
N1	0.4009 (7)	1.1081 (5)	0.2832 (3)	0.0524 (10)	
N2	0.3572 (7)	0.8821 (6)	0.3839 (3)	0.0624 (12)	
N3	0.4550 (7)	0.7685 (6)	0.4514 (3)	0.0615 (12)	
C1	0.1565 (11)	0.6619 (9)	−0.0019 (4)	0.084 (2)	
H1A	0.2071	0.5921	−0.0410	0.126*	
H1B	0.0557	0.7626	−0.0281	0.126*	
H1C	0.0839	0.6105	0.0462	0.126*	
C2	0.2971 (9)	0.7920 (7)	0.0792 (4)	0.0628 (14)	
C3	0.0998 (10)	0.8504 (8)	0.1184 (4)	0.0731 (16)	
H3B	−0.0205	0.8231	0.1084	0.088*	
C4	0.0759 (9)	0.9502 (8)	0.1733 (4)	0.0687 (16)	
H4A	−0.0604	0.9869	0.2006	0.082*	
C5	0.2469 (8)	0.9971 (7)	0.1889 (3)	0.0550 (12)	
C6	0.4424 (11)	0.9355 (10)	0.1499 (5)	0.107 (3)	
H6A	0.5630	0.9621	0.1602	0.128*	
C7	0.4704 (12)	0.8350 (10)	0.0956 (5)	0.109	
H7A	0.6079	0.7959	0.0697	0.131*	
C8	0.2051 (8)	1.1190 (6)	0.2422 (3)	0.0544 (13)	
H8A	0.0890	1.1042	0.2852	0.065*	
C9	0.3907 (10)	1.3489 (8)	0.1783 (4)	0.0770 (18)	
H9A	0.4707	1.3304	0.1258	0.092*	
H9B	0.3797	1.4573	0.1835	0.092*	
C10	0.5063 (9)	1.2245 (7)	0.2508 (3)	0.0567 (13)	
C11	0.4717 (8)	0.9827 (6)	0.3532 (3)	0.0505 (12)	
C12	0.6418 (8)	0.7842 (6)	0.4677 (3)	0.0470 (11)	
C13	0.7762 (8)	0.6774 (6)	0.5357 (3)	0.0480 (11)	
C14	0.9628 (9)	0.7058 (7)	0.5547 (3)	0.0608 (14)	
H14A	1.0009	0.7966	0.5243	0.073*	
C15	1.0908 (10)	0.6009 (7)	0.6181 (4)	0.0668 (15)	
H15A	1.2114	0.6223	0.6324	0.080*	
C16	1.0368 (9)	0.4640 (7)	0.6596 (3)	0.0613 (15)	
C17	0.8542 (10)	0.4310 (7)	0.6425 (3)	0.0647 (15)	
H17A	0.8189	0.3390	0.6728	0.078*	
C18	0.7262 (9)	0.5372 (6)	0.5799 (3)	0.0584 (14)	
H18A	0.6044	0.5156	0.5668	0.070*	

### Atomic displacement parameters (Å^2^)

**Table:**

	*U*^11^	*U*^22^	*U*^33^	*U*^12^	*U*^13^	*U*^23^
S1	0.0724 (10)	0.0617 (9)	0.0743 (10)	−0.0157 (8)	−0.0163 (8)	−0.0123 (8)
S2	0.0476 (7)	0.0587 (8)	0.0565 (8)	−0.0219 (6)	−0.0011 (6)	−0.0152 (6)
F	0.092 (3)	0.081 (2)	0.075 (2)	−0.008 (2)	−0.022 (2)	−0.0116 (19)
O1	0.085 (3)	0.102 (3)	0.098 (3)	−0.038 (3)	0.013 (3)	−0.059 (3)
O2	0.054 (2)	0.069 (3)	0.081 (3)	−0.0299 (19)	−0.005 (2)	−0.008 (2)
N1	0.052 (2)	0.056 (3)	0.056 (3)	−0.023 (2)	0.000 (2)	−0.017 (2)
N2	0.057 (3)	0.064 (3)	0.069 (3)	−0.029 (2)	−0.007 (2)	−0.003 (2)
N3	0.058 (3)	0.068 (3)	0.063 (3)	−0.028 (2)	−0.007 (2)	−0.008 (2)
C1	0.100 (5)	0.094 (5)	0.075 (4)	−0.038 (4)	−0.012 (4)	−0.032 (4)
C2	0.064 (3)	0.061 (3)	0.067 (3)	−0.015 (3)	−0.002 (3)	−0.027 (3)
C3	0.064 (3)	0.089 (4)	0.077 (4)	−0.026 (3)	0.003 (3)	−0.038 (3)
C4	0.051 (3)	0.090 (4)	0.072 (4)	−0.024 (3)	0.011 (3)	−0.035 (3)
C5	0.050 (3)	0.062 (3)	0.054 (3)	−0.016 (2)	−0.004 (2)	−0.014 (2)
C6	0.062 (4)	0.139 (6)	0.152 (6)	−0.034 (4)	0.011 (4)	−0.096 (5)
C7	0.070	0.148	0.146	−0.040	0.021	−0.105
C8	0.046 (3)	0.059 (3)	0.061 (3)	−0.018 (2)	−0.003 (2)	−0.016 (3)
C9	0.083 (4)	0.081 (4)	0.065 (4)	−0.037 (4)	0.005 (3)	−0.001 (3)
C10	0.055 (3)	0.061 (3)	0.058 (3)	−0.024 (3)	0.008 (3)	−0.017 (3)
C11	0.049 (3)	0.057 (3)	0.051 (3)	−0.021 (2)	0.004 (2)	−0.018 (2)
C12	0.046 (3)	0.047 (3)	0.047 (3)	−0.013 (2)	0.005 (2)	−0.014 (2)
C13	0.042 (3)	0.050 (3)	0.048 (3)	−0.007 (2)	0.006 (2)	−0.016 (2)
C14	0.060 (3)	0.072 (4)	0.057 (3)	−0.028 (3)	0.001 (3)	−0.016 (3)
C15	0.065 (4)	0.075 (4)	0.061 (4)	−0.014 (3)	−0.015 (3)	−0.019 (3)
C16	0.065 (4)	0.059 (3)	0.045 (3)	0.003 (3)	−0.012 (3)	−0.008 (3)
C17	0.074 (4)	0.058 (3)	0.058 (3)	−0.018 (3)	−0.004 (3)	−0.005 (3)
C18	0.060 (3)	0.059 (3)	0.063 (3)	−0.027 (3)	0.000 (3)	−0.017 (3)

### Geometric parameters (Å, °)

**Table:**

S1—C9	1.790 (6)	C4—H4A	0.9300
S1—C8	1.828 (5)	C5—C6	1.349 (8)
S2—C11	1.716 (5)	C5—C8	1.497 (7)
S2—C12	1.741 (5)	C6—C7	1.373 (9)
F—C16	1.359 (6)	C6—H6A	0.9300
O1—C2	1.368 (6)	C7—H7A	0.9300
O1—C1	1.428 (7)	C8—H8A	0.9800
O2—C10	1.221 (6)	C9—C10	1.504 (8)
N1—C10	1.384 (6)	C9—H9A	0.9700
N1—C11	1.402 (6)	C9—H9B	0.9700
N1—C8	1.475 (6)	C12—C13	1.454 (7)
N2—C11	1.309 (6)	C13—C14	1.396 (7)
N2—N3	1.371 (6)	C13—C18	1.396 (7)
N3—C12	1.326 (6)	C14—C15	1.377 (8)
C1—H1A	0.9600	C14—H14A	0.9300
C1—H1B	0.9600	C15—C16	1.373 (8)
C1—H1C	0.9600	C15—H15A	0.9300
C2—C3	1.354 (8)	C16—C17	1.381 (8)
C2—C7	1.371 (8)	C17—C18	1.372 (7)
C3—C4	1.380 (8)	C17—H17A	0.9300
C3—H3B	0.9300	C18—H18A	0.9300
C4—C5	1.373 (7)		
			
C9—S1—C8	93.5 (3)	C5—C8—H8A	109.0
C11—S2—C12	85.8 (2)	S1—C8—H8A	109.0
C2—O1—C1	117.6 (5)	C10—C9—S1	106.9 (4)
C10—N1—C11	122.7 (4)	C10—C9—H9A	110.3
C10—N1—C8	118.0 (4)	S1—C9—H9A	110.3
C11—N1—C8	119.3 (4)	C10—C9—H9B	110.3
C11—N2—N3	110.4 (4)	S1—C9—H9B	110.3
C12—N3—N2	113.4 (4)	H9A—C9—H9B	108.6
O1—C1—H1A	109.5	O2—C10—N1	122.5 (5)
O1—C1—H1B	109.5	O2—C10—C9	125.7 (5)
H1A—C1—H1B	109.5	N1—C10—C9	111.7 (5)
O1—C1—H1C	109.5	N2—C11—N1	119.7 (4)
H1A—C1—H1C	109.5	N2—C11—S2	116.8 (4)
H1B—C1—H1C	109.5	N1—C11—S2	123.4 (4)
C3—C2—O1	125.2 (5)	N3—C12—C13	123.5 (5)
C3—C2—C7	118.1 (6)	N3—C12—S2	113.5 (4)
O1—C2—C7	116.7 (5)	C13—C12—S2	123.0 (4)
C2—C3—C4	120.4 (6)	C14—C13—C18	118.8 (5)
C2—C3—H3B	119.8	C14—C13—C12	121.6 (5)
C4—C3—H3B	119.8	C18—C13—C12	119.5 (5)
C5—C4—C3	122.1 (5)	C15—C14—C13	120.7 (5)
C5—C4—H4A	118.9	C15—C14—H14A	119.7
C3—C4—H4A	118.9	C13—C14—H14A	119.7
C6—C5—C4	116.3 (5)	C16—C15—C14	118.6 (5)
C6—C5—C8	124.1 (5)	C16—C15—H15A	120.7
C4—C5—C8	119.5 (5)	C14—C15—H15A	120.7
C5—C6—C7	122.5 (6)	F—C16—C15	118.2 (5)
C5—C6—H6A	118.7	F—C16—C17	119.3 (6)
C7—C6—H6A	118.7	C15—C16—C17	122.5 (5)
C2—C7—C6	120.5 (6)	C18—C17—C16	118.5 (5)
C2—C7—H7A	119.7	C18—C17—H17A	120.8
C6—C7—H7A	119.7	C16—C17—H17A	120.8
N1—C8—C5	113.5 (4)	C17—C18—C13	120.9 (5)
N1—C8—S1	103.5 (3)	C17—C18—H18A	119.6
C5—C8—S1	112.5 (4)	C13—C18—H18A	119.6
N1—C8—H8A	109.0		
			
C11—N2—N3—C12	−1.9 (6)	S1—C9—C10—O2	−168.3 (5)
C1—O1—C2—C3	7.2 (9)	S1—C9—C10—N1	15.5 (6)
C1—O1—C2—C7	−172.7 (7)	N3—N2—C11—N1	179.5 (4)
O1—C2—C3—C4	−180.0 (6)	N3—N2—C11—S2	1.2 (6)
C7—C2—C3—C4	0.0 (10)	C10—N1—C11—N2	175.9 (5)
C2—C3—C4—C5	1.5 (10)	C8—N1—C11—N2	−3.5 (7)
C3—C4—C5—C6	−2.3 (10)	C10—N1—C11—S2	−5.9 (7)
C3—C4—C5—C8	173.7 (6)	C8—N1—C11—S2	174.7 (4)
C4—C5—C6—C7	1.8 (12)	C12—S2—C11—N2	−0.2 (4)
C8—C5—C6—C7	−174.0 (7)	C12—S2—C11—N1	−178.4 (4)
C3—C2—C7—C6	−0.5 (12)	N2—N3—C12—C13	−179.5 (4)
O1—C2—C7—C6	179.5 (8)	N2—N3—C12—S2	1.8 (6)
C5—C6—C7—C2	−0.4 (14)	C11—S2—C12—N3	−0.9 (4)
C10—N1—C8—C5	104.3 (5)	C11—S2—C12—C13	−179.7 (4)
C11—N1—C8—C5	−76.2 (6)	N3—C12—C13—C14	−174.9 (5)
C10—N1—C8—S1	−17.9 (5)	S2—C12—C13—C14	3.7 (7)
C11—N1—C8—S1	161.6 (4)	N3—C12—C13—C18	9.1 (7)
C6—C5—C8—N1	−28.9 (9)	S2—C12—C13—C18	−172.3 (4)
C4—C5—C8—N1	155.4 (5)	C18—C13—C14—C15	−2.4 (8)
C6—C5—C8—S1	88.3 (7)	C12—C13—C14—C15	−178.4 (5)
C4—C5—C8—S1	−87.4 (6)	C13—C14—C15—C16	2.8 (8)
C9—S1—C8—N1	22.4 (4)	C14—C15—C16—F	177.9 (5)
C9—S1—C8—C5	−100.6 (4)	C14—C15—C16—C17	−2.6 (9)
C8—S1—C9—C10	−22.2 (5)	F—C16—C17—C18	−178.7 (5)
C11—N1—C10—O2	6.3 (8)	C15—C16—C17—C18	1.9 (9)
C8—N1—C10—O2	−174.3 (5)	C16—C17—C18—C13	−1.4 (8)
C11—N1—C10—C9	−177.4 (5)	C14—C13—C18—C17	1.6 (8)
C8—N1—C10—C9	2.1 (7)	C12—C13—C18—C17	177.7 (5)

### Hydrogen-bond geometry (Å, °)

**Table:**

*D*—H···*A*	*D*—H	H···*A*	*D*···*A*	*D*—H···*A*
C6—H6A···N1	0.93	2.60	2.917 (9)	101
C8—H8A···O2^i^	0.98	2.52	3.233 (7)	129
C14—H14A···S2	0.93	2.74	3.146 (6)	107
C18—H18A···N3	0.93	2.57	2.881 (7)	100

Symmetry codes: (i) *x*−1, *y*, *z*.
